# Vitamin D_3_ and 25-hydroxyvitamin D_3_ in pork and their relationship to vitamin D status in pigs

**DOI:** 10.1017/jns.2015.28

**Published:** 2016-01-08

**Authors:** Anders Burild, Charlotte Lauridsen, Nasrin Faqir, Helle M. Sommer, Jette Jakobsen

**Affiliations:** 1 National Food Institute, Technical University of Denmark, Mørkhøj Bygade 19, 2860 Søborg, Denmark; 2 Department of Animal Science, Aarhus University, Blichers Alle 20, 8830 Tjele, Denmark; 3 Department of Applied Mathematics and Computer Science, Technical University of Denmark, 2800 Lyngby, Denmark

**Keywords:** Vitamin D_3_, 25-Hydroxyvitamin D_3_, Biofortification, Bioavailability, 25(OH)D_3_, 25-hydroxyvitamin D_3_

## Abstract

The content of vitamin D in pork produced in conventional systems depends on the vitamin D concentration in the pig feed. Both vitamin D_3_ and 25-hydroxyvitamin D_3_ (25(OH)D_3_) are essential sources of dietary vitamin D; however, bioavailability assessed by serum 25(OH)D_3_ concentration is reported to be different between the two sources. Furthermore, the relationship between serum 25(OH)D_3_ level and the tissue content of vitamin D_3_ and 25(OH)D_3_ is unknown. The objective of this study was to investigate the potential of increasing the content of vitamin D in different pig tissues by increasing the levels of vitamin D_3_ and 25(OH)D_3_ in the pig feed for 49 d before slaughter. Concurrently, the 25(OH)D_3_ level in serum was investigated as a biomarker to assess the content of vitamin D_3_ and 25(OH)D_3_ in pig tissues. Adipose tissue, white and red muscle, the liver and serum were sampled from pigs fed feed containing either vitamin D_3_ or 25(OH)D_3_ at 5, 20, 35 or 50 µg/kg feed for 7 weeks before slaughter. The tissue 25(OH)D_3_ level was significantly higher in the pigs fed 25(OH)D_3_ compared with those fed vitamin D_3_, while the tissue vitamin D_3_ level was higher in the pigs fed vitamin D_3_ compared with those fed 25(OH)D_3_. The content of 25(OH)D_3_ in the different tissues fully correlated with the serum 25(OH)D_3_ level, whereas the correlation between the tissue content of vitamin D_3_ and serum 25(OH)D_3_ was dependent on the source of the ingested vitamin D_3_.

Vitamin D belongs to the group of lipophilic vitamins and accumulates in the adipose tissue of rats, pigs and humans^(^^1^^–^^3^^)^. Serum or plasma 25-hydroxyvitamin D is considered the best biomarker of vitamin D status^(^^4^^)^, but its correlation with the tissue concentration of vitamin D is unknown^(^^5^^)^.

Vitamin D exists in two major forms: vitamin D_2_ and vitamin D_3_. Vitamin D_3_ is synthesised in the skin after UV exposure^(^^6^^)^ and is naturally found as vitamin D_3_ and 25-hydroxyvitamin D_3_ (25(OH)D_3_) in products of animal origin, e.g. meat and eggs^(^^7^^,^^8^^)^. Within the European Union, vitamin D_3_ is the main vitamin D source in animal feed, but recently, 25(OH)D_3_ has been approved for supplementary use in pigs and hens. The maximum permitted level of vitamin D sources is 50 µg/kg pig feed^(^^9^^,^^10^^)^.

Comparing the serum and plasma 25(OH)D_3_ levels after the administration of either of the two sources of vitamin D_3_, oral 25(OH)D_3_ has been found to be more potent than oral vitamin D_3_, although the data are inconsistent. In humans, oral 25(OH)D_3_ is 2- to 5-fold more potent than oral vitamin D_3_^(^^11^^,^^12^^)^; and in pigs, oral 25(OH)D_3_ is 1- to 3-fold more potent than oral vitamin D_3_^(^^2^^,^^13^^–^^15^^)^.

For humans 25(OH)D_3_ is a significant compound for the dietary intake of vitamin D^(^^16^^)^. However, the vitamin D source, either vitamin D_3_ or 25(OH)D_3_, used in pig feed determines distribution of the two vitamin D metabolites in the fat in slaughter pigs fed the same feed throughout their lives^(^^2^^)^. Moreover, the amount of vitamin D_3_ stored depends on the fat content of the given tissue^(^^2^^,^^17^^)^. However, information regarding the difference between the distribution of oral 25(OH)D_3_ and oral vitamin D_3_ stored in the body is sparse. Since 25(OH)D_3_ is more polar than vitamin D_3_ and the affinity of 25(OH)D_3_ for vitamin D-binding protein is more than 500 times stronger than that of vitamin D_3_, more vitamin D_3_ is assumed to be present in its free form, which will allow it to diffuse directly into adjacent tissues^(^^18^^)^.

The aims of this study were to investigate the concentrations of vitamin D_3_ and 25(OH)D_3_ in pork meat (muscle, adipose and liver tissue) from pigs that were fed different doses of vitamin D_3_ or 25(OH)D_3_, and to assess whether serum 25(OH)D_3_ is a biomarker for the tissue concentration of vitamin D_3_ and 25(OH)D_3_.

## Materials and methods

### Animal experiment

Details of the animal study including diet composition can be obtained in Lauridsen *et al*.^(^^13^^)^. In brief, 160 prepubertal gilts ((Danish Landrace × Danish Yorkshire) and Duroc; body weight 156 (se = 7·1) kg) were included in the main pig study investigating the nutritional benefits of vitamin D for reproducing female pigs, with special emphasis on bioefficiency of the two vitamin D sources in terms of bioavailability, early reproduction, bone status markers, and transfer of vitamin D to the progeny^(^^13^^)^. The animals were randomly assigned to dietary treatments containing four concentrations (5, 20, 35 and 50 µg/kg feed) of one of the two different vitamin D sources: vitamin D_3_ and 25(OH)D_3_. A subpopulation of four animals from each of the eight dietary treatments, except three from the group receiving 50 µg vitamin D_3_ and five from the group receiving 35 µg 25(OH)D_3_/kg (total *n* = 32 pigs, body weight = 186 kg), was randomly selected from the population of 160 gilts (body weight 186 (se = 7·2) kg at slaughter). The basal diet consisted of barley (75 %), soyabean meal (8 %), wheat bran (5 %), green grass (5 %), molasses (3 %), animal fat (2 %), and vitamins and minerals (2 %). The analysed concentrations in the diets were very close to the formulated amounts^(^^13^^)^. The study was carried out at Aarhus University, Foulum, Denmark, and the pigs were housed indoors with an artificial lighting (no UVB) regimen 12 h per d. The pigs were killed after 49 d on the experimental diets.

### Sampling

Blood samples obtained at slaughter from the *vena jugularis* were collected in Vacutainer tubes containing no additives and processed to serum, which was immediately stored at −80°C until analysis. After the carcasses were eviscerated, samples of the liver, *longisimus dorsi* (loin), and of the *psoas major* (red muscle tissue) were obtained. Adipose tissue and muscle tissue (white muscle tissue) were carefully dissected from the loin. All samples were stored in plastic bags at −20°C until analysis. Before analysis each sample was slowly thawed and homogenised for 2 min (1094 Homogenizer; Tecator).

### Analysis of 25-hydroxyvitamin D_3_ and vitamin D_3_ in tissue and 25-hydroxyvitamin D_3_ in serum and tissues

The tissue samples were analysed by a previous published method using HPLC^(^^2^^,^^19^^)^. In short, the internal standard of vitamin D_2_ and 25(OH)D_2_ were added to the test sample. The samples were saponified, liquid/liquid extracted, cleaned-up in a solid-phase step, followed by a preparative normal-phase HPLC step. For the final separation, detection and quantification reversed-phase chromatography coupled to a UV detector and a diode array detector (DAD) was used. The analyses were performed accredited according to ISO17025^(^^20^^)^, and quality control included participation in proficiency testing (FAPAS; www.fapas.com). Duplicate analyses were used to assess precision, which for samples with contents of vitamin D_3_ and 25(OH)D_3_ above 1 µg/kg showed a between-day precision of ≤5·6 and ≤5·1 %, respectively. For samples with contents below 1 µg/kg the between-day precision was ≤0·06 µg/kg for both compounds.

Serum was analysed for 25(OH)D_3_ by HPLC equipped with a DAD and a UV detector for detection and quantification as described in detail elsewhere^(^^21^^)^, showing a between-day precision of ≤5·7 % from duplicate analyses. Participation in the Vitamin D External Quality Assessment Scheme (DEQAS; Charing Cross Hospital, London, UK) ensured trueness of the results. Samples of liver and red muscles were analysed for the groups fed 5 or 50 µg/kg feed only, due to economic resources.

### Analysis of fat content in muscle tissue

The content of fat in the muscle tissue was determined by the gravimetric method by a modified Schmid–Bondzynski–Ratslaff (SBR) method^(^^22^^)^. In short, the sample was boiled with hydrochloric acid followed by the addition of ethanol and extraction of the lipids with diethyl ether–petroleum ether (1:1, v/v). After evaporation of the solvent, the fat was weighed. Due to a limited amount of sample of the red muscle, fat content was only determined on samples from pigs receiving 20 or 35 µg/kg feed.

### Statistics

The effects of vitamin D_3_ form (vitamin D_3_, 25(OH)D_3_) and level in feed (5, 20, 35, 50 µg/kg) on the content of 25(OH)D_3_ and vitamin D_3_ in tissues were analysed by the regression model:
1
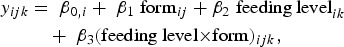

where ‘*y*’ is the response variable for eight different types of measurements (*i* = 1,2,…,8) of vitamin D, i.e. the combination of measured vitamin D_3_ or 25(OH)D_3_ in four different meat cuts: adipose tissue, white muscle tissue, red muscle tissue, and liver. The index ‘*j*’ (= 1, 2) is used for the two forms of vitamin D, vitamin D_3_ and 25(OH)D_3_, in the feed. For each type of response variable (*i*) β_0_ refers to the intercept, β_1*j*_ is a categorical parameter, and β_2_and β_3*j*_ are regressor parameters.

β_0_ and β_1*j*_ represent the cut offs and β_2_ and β_3*j*_ represent the slopes of the regression lines for feeding level of vitamin D_3_ and 25(OH)D_3_, respectively. The two regression lines for each response variable were analysed simultaneously. This way not only the power of the test was increased by increased df, the simultaneously estimation also served the purpose of being able to determine whether the two cut-offs (β_1*j*_) were significantly different from each other, and to determine whether the slopes (β_3*j*_) were significantly different from each other.

The associations between serum 25(OH)D_3_, dietary vitamin D form, and vitamin D_3_ and 25(OH)D_3_ in tissues were investigated by a similar type of model as shown above (equation 1), except that the explanatory variable ‘feeding level’ was replaced by ‘serum’.

In the dataset one outlier was detected using the methods described by Cook & Weisberg^(^^23^^)^. Results in the tables and figures are given as means with their standard errors. All data were analysed using proc glm, SAS version 9.3 (SAS Institute), and a significant level of α = 5 % was used as cut-off value for the *P* values. For plotting, the program Prism 5 for Windows (GraphPad Software) was used.

### Ethics statement

The experiment complied with the guidelines of the Danish Ministry of Justice with respect to animal experimentation and care of animals under study.

## Results

All results (means with their standard errors) of the content of vitamin D_3_ and 25(OH)D_3_ in meat cuts, i.e. adipose tissue, white muscle tissue, red muscle tissue and liver, and serum 25(OH)D_3_ are shown in Tables 1 and 2. Overall, the content of 25(OH)D_3_ in serum was between 8·7 and 67·1 ng/ml. In the tissues the content of 25(OH)D_3_ was between 0·37 and 11·9 µg/kg, while the content of vitamin D_3_ was between 0·10 and 8·41 µg/kg.

**Table 1.** Serum level and content of vitamin D_3_ and 25-hydroxyvitamin D_3_ (25(OH)D_3_) in adipose tissue (subcutaneous fat from loin), white muscle tissue (lean meat from loin), red muscle tissue (chain muscle) and in the liver following feeding for 49 d with 5, 20, 35 and 50 µg vitamin D_3_/kg feed (*n* 4, 4, 4, 3, respectively) (Mean values with their standard errors)

Vitamin D_3_ in feed (μg/kg feed)…	5		20		35		50	
	Mean	sem	Mean	sem	Mean	sem	Mean	sem
Serum								
25(OH)D_3_ (ng/ml)*	8·7	1·5	12·2	1·5	21·7	1·3	27·2	1·7
Adipose tissue								
Vitamin D_3_ (μg/kg)*†	2·08	0·53	2·78	0·13	5·45	0·62	7·59	0·39
25(OH)D_3_ (μg/kg)*	0·86	0·10	1·03	0·15	1·29	0·15	2·03	0·10
White muscle tissue								
Vitamin D_3_ (μg/kg)*†	0·20	0·04	0·46	0·05	0·69	0·06	1·18	0·10
25(OH)D_3_ (μg/kg)*	0·37	0·04	0·53	0·02	0·66	0·04	1·07	0·19
Red muscle tissue								
Vitamin D_3_ (μg/kg)*†	0·49	0·15	N/A		N/A		2·50	0·26
25(OH)D_3_ (μg/kg)*	0·54	0·18	N/A		N/A		1·81	0·24
Liver								
Vitamin D_3_ (μg/kg)*†	0·68	0·07	N/A		N/A		8·41	1·36
25(OH)D_3_ (μg/kg)*	1·33	0·05	N/A		N/A		4·52	0·36

N/A, not applicable.

* Increasing doses of vitamin D_3_ in the feed increased the content of vitamin D_3_ and 25(OH)D_3_ (*P* < 0·001).

† The vitamin D_3_ content was significantly higher (*P* < 0·001) for pigs fed vitamin D_3_ compared with 25(OH)D_3_ in the feed (data shown in Table 2).

**Table 2.** Serum level and content of vitamin D_3_ and 25-hydroxyvitamin D_3_ (25(OH)D_3_) in adipose tissue (subcutaneous fat from loin), white muscle tissue (lean meat from loin), red muscle tissue (chain muscle) and in the liver following feeding for 49 d with 5, 20, 35 and 50 µg of 25(OH)D_3_/kg of feed (*n* 4, 4, 5, 4, respectively) (Mean values with their standard errors)

25(OH)D_3_ in feed (μg/kg feed)…	5		20		35		50	
	Mean	sem	Mean	sem	Mean	sem	Mean	sem
Serum								
25(OH)D_3_ (ng/ml)*	11·4	1·8	31·1	2·8	49·2	6·1	67·1	9·4
Adipose tissue								
Vitamin D_3_ (μg/kg)*†	1·51	0·28	2·71	0·54	3·37	1·05	3·25	0·59
25(OH)D_3_ (μg/kg)*	0·95	0·17	1·42	0·16	3·09	0·53	4·15	0·83
White muscle tissue								
Vitamin D_3_ (μg/kg)*†	0·10	0·03	0·12	0·02	0·46	0·28	0·17	0·01
25(OH)D_3_ (μg/kg)*	0·41	0·05	1·14	0·09	1·85	0·25	2·81	0·32
Red muscle tissue								
Vitamin D_3_ (μg/kg)*†	0·34	0·04	N/A		N/A		0·45	0·08
25(OH)D_3_ (μg/kg)*	0·67	0·07	N/A		N/A		4·02	0·68
Liver								
Vitamin D_3_ (μg/kg)*†	0·20	0·02	N/A		N/A		0·40	0·07
25(OH)D_3_ (μg/kg)*	2·74	0·31	N/A		N/A		11·9	1·96

N/A, not applicable.

* Increasing doses of 25(OH)D_3_ in the feed increased the content of vitamin D_3_ and 25(OH)D_3_ (*P* < 0·001).

† The 25(OH)D_3_ content was significantly higher (*P* < 0·002) for pigs fed 25(OH)D_3_ compared with vitamin D_3_ in the feed (data shown in Table 1).

The content of fat in red muscle tissue and in white muscle tissues was 3·7 (sem 1·2) % (*n* = 32) and 2·1 (sem 0·7) % (*n* = 16), respectively.

**Fig. 1.**
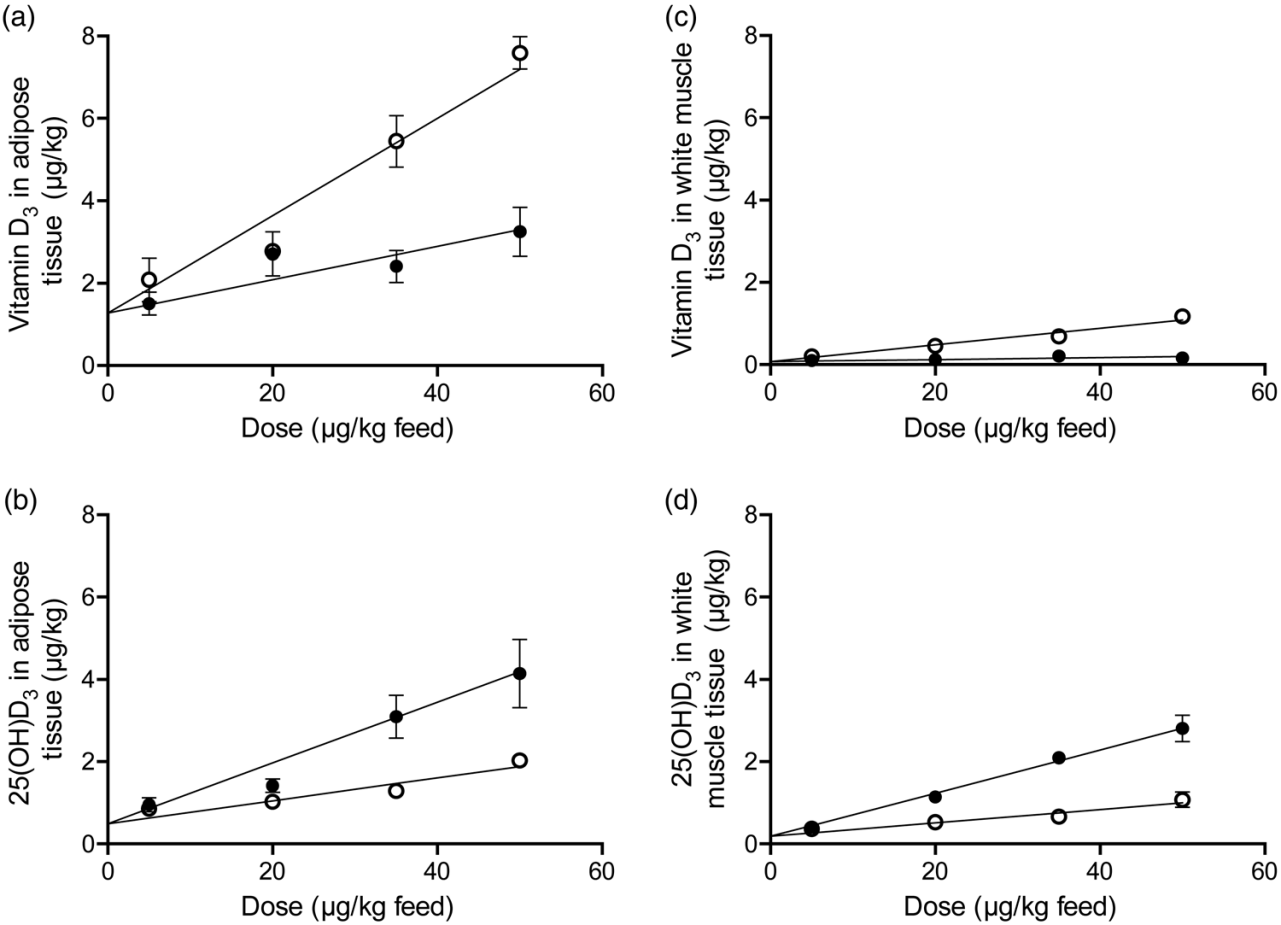
Vitamin D_3_ (○) (a and c) and 25-hydroxyvitamin D_3_ (25(OH)D_3_; ●) (b and d) in adipose tissue and white muscle tissue plotted against content of vitamin D_3_ or 25(OH)D_3_ in feed. Values are means, with standard errors represented by vertical bars.

Overall, for all eight types of response variables the increasing doses of either vitamin D_3_ or 25(OH)D_3_ in feed increased the content of 25(OH)D_3_ and vitamin D_3_ in all tissues (β_2_: *P* < 0·001). The vitamin D_3_ content in all analysed tissues was significantly (β_3_: *P* < 0·001) higher for pigs fed vitamin D_3_, whereas the tissue content of 25(OH)D_3_ was significantly higher (β_3_: *P* < 0·002) in all tissues when 25(OH)D_3_ was provided in the feed. ‘Dose 0’ is a theoretical result for vitamin D in tissues if concentration of vitamin D in the feed is zero. The parameter β_1_ was not significant for any of the analyses meaning that for ‘dose 0’ the content of vitamin D_3_ and 25(OH)D_3_ in all tissues was the same for the two regression lines (Figs 1(a)–(d)). The baseline values for the two groups are thus the same.

**Fig. 2.**
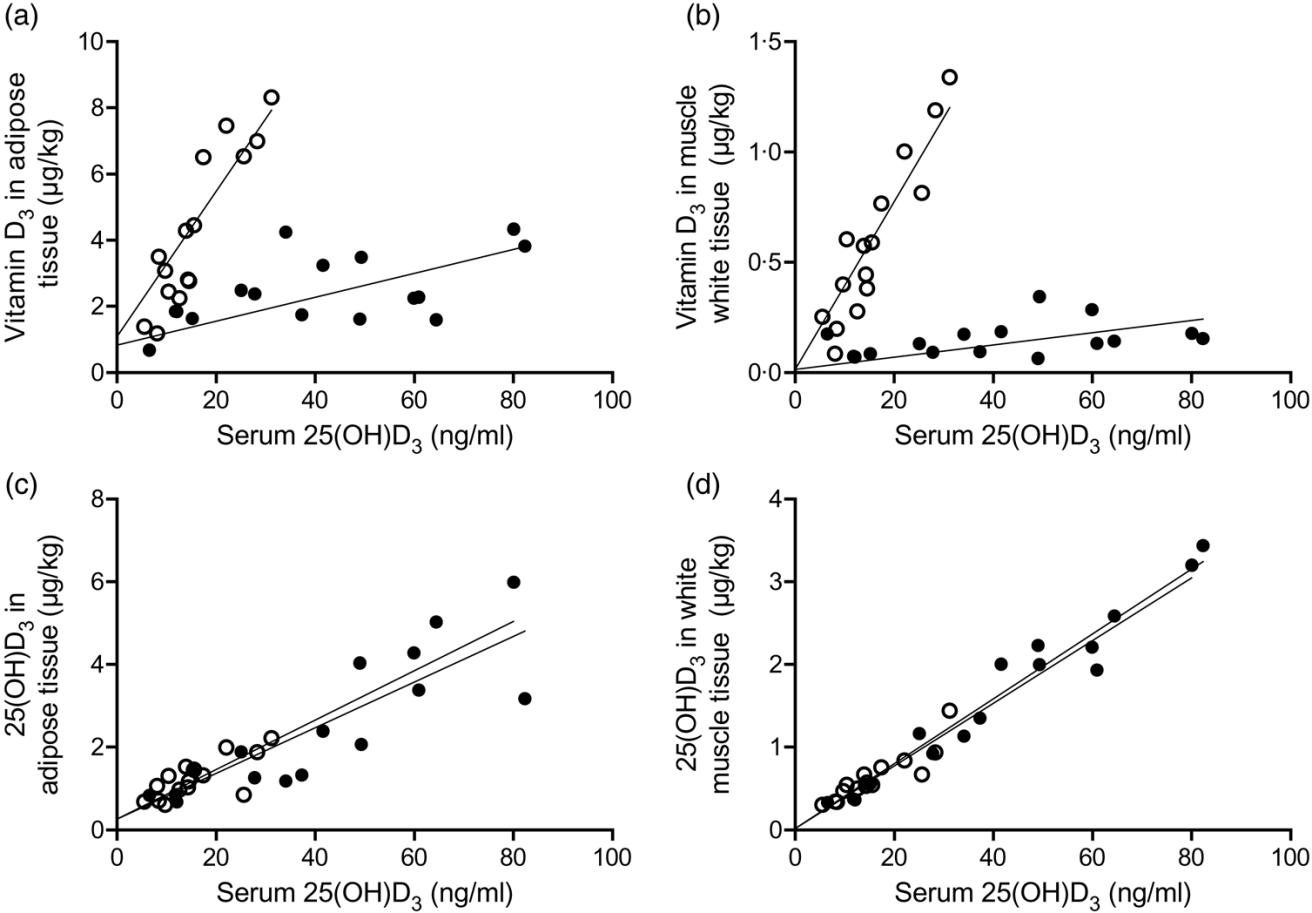
Content of vitamin D_3_ in adipose and white muscle tissues *v.* serum 25-hydroxyvitamin D_3_ (25(OH)D_3_) (a and b) were linearly correlated (*P* < 0·001), and concentration dependent on the dietary vitamin D_3_ form (*P* < 0·001). Content of 25(OH)D_3_ in adipose and muscle tissues *v.* serum 25(OH)D_3_ (c and d) for pigs fed either vitamin D_3_ or 25(OH)D_3_ were linearly correlated (*P* < 0·001), but concentration independent on the dietary vitamin D_3_ form (*P* > 0·72). (○), Vitamin D_3_ in feed; (●), 25(OH)D_3_ in feed.

As shown in Figs 2(a) and (b), the content of vitamin D_3_ in adipose and white muscle tissues was linearly correlated with serum 25(OH)D_3_ (β_2_: *P* < 0·001). Furthermore, the concentration was dependent on the dietary vitamin D_3_ form, as the interaction term was significant (β_3_: *P* < 0·001).

The content of 25(OH)D_3_ in adipose and white muscle tissues (Figs 2(c) and (d)) was also linearly correlated with serum 25(OH)D_3_ (β_2_: *P* < 0·001). The correlation coefficient was, however, independent of the dietary vitamin D_3_ form (β_3_: *P* > 0·72).

## Discussion

In the present study, we compared the effects of different levels of 25(OH)D_3_ and vitamin D_3_ in pig feed on the concentrations of both 25(OH)D_3_ and vitamin D_3_ in different pig tissues. Adipose, liver and muscle tissues were chosen for the analysis as representative of the major tissues in the pig that are consumed by humans.

Jakobsen *et al*.^(^^2^^)^ found that vitamin D_3_ and 25(OH)D_3_ levels depend on the fat content. In the present study we found a higher content of vitamin D in adipose tissue than in muscle tissue. We presume that the difference between red muscle tissue compared with white muscle tissue is due to the difference in fat content.

Other studies in pigs have investigated a single feeding level of each of the two vitamin D metabolites on their tissue contents^(^^2^^,^^15^^)^. Comparing absolute levels obtained in different studies has to be done with caution, due to differences in study design, experimental animals and analytical methods.

The pigs fed 50 µg vitamin D_3_/kg feed had vitamin D_3_ levels of 7·59 µg/kg in adipose tissue (subcutaneous fat) and 1·18 µg/kg in white muscle tissue (lean meat). These levels are in the same range as pigs fed 24 µg vitamin D_3_/kg feed for 70 d resulting in 7·47 µg vitamin D_3_/kg in adipose tissue and 1·11 µg vitamin D_3_/kg in white muscle tissue, respectively^(^^2^^)^. The corresponding concentrations of 25(OH)D_3_ in subcutaneous fat and lean meat we found to be at 2·03 and 1·07 µg/kg, respectively, were also similar to previously found^(^^2^^)^. Although the amount of vitamin D_3_ in the feed of the two studies was different, 50 µg/kg feed and 24 µg/kg feed, the levels of vitamin D_3_ and 25(OH)D_3_ in the tissues were similar. Another study also provided 50 µg vitamin D_3_/kg feed to slaughter pigs, and reported no tissue content of 25(OH)D_3_^(^^15^^)^; however, the method used showed a limit of quantification of 5 µg/kg, thus excluding quantification at the levels that we report.

The pigs (females) in the present study provided with 20 µg of 25(OH)D_3_/kg feed had similar levels of 25(OH)D_3_ in adipose tissue and lean meat as slaughter pigs (both sexes) fed 20 µg of 25(OH)D_3_/kg feed^(^^2^^)^. In contrast, the corresponding level of vitamin D_3_ was five times higher in the pigs in the present study: 2·71 *v.* 0·57 µg/kg. This discrepancy may be due to differences in study design. In the present study, all pigs were fed vitamin D_3_ up to inclusion in the study, while the pigs were divided into the different feeding groups after weaning^(^^2^^)^. Höller *et al*.^(^^15^^)^ reported a content of 5·7 µg of 25(OH)D_3_/kg lean meat of slaughter pigs fed 50 µg of 25(OH)D_3_/kg feed for 119 d. This value is 50–100 % higher than we found in white and red muscle tissue.

Interestingly, the vitamin D level found in the present study was relatively low compared with the vitamin D content determined in a study of Göttingen minipigs^(^^24^^)^. The vitamin D_3_ levels in the adipose tissues of the minipigs were 98 µg vitamin D_3_/kg and 67 µg of 25(OH)D_3_/kg, i.e. more than 10 times as high as found in the present study (7·59 and 2·03 μg/kg, respectively). The minipigs were fed 3·7–4·4 µg vitamin D_3_/kg body weight (approximately 200 µg/kg feed) for 5 weeks, which is about four times higher than the highest level in the present study. However, it can be speculated that the content of fat in the different breeds affects the concentration of vitamin D, e.g. the Göttingen minipig is a breed with small stores of fat, which will result in a higher concentration in the fat if the same amount of vitamin D is stored.

Our study showed that increased vitamin D_3_ levels in feed increase the contents of vitamin D_3_ and 25(OH)D_3_ in pork meat, and increased 25(OH)D_3_ levels in feed increase the content of 25(OH)D_3_ in pork. These findings demonstrate that increasing the levels of vitamin D in pig feed has the potential to increase the dietary intake of vitamin D from biofortified pork; however, the potential has a limitation due to the maximum level of vitamin D allowed in feed^(^^9^^,^^10^^)^.

Furthermore, we demonstrated that the adipose and white muscle tissue content of 25(OH)D_3_ could be predicted from the serum 25(OH)D_3_ level independently of the ingested form of vitamin D_3_. The content of vitamin D_3_ in these tissues was also related to the serum 25(OH)D_3_ level, but the correlation depended on the dietary source of vitamin D_3_.

Extrapolation from pig data has estimated the level of vitamin D stores in humans^(^^25^^)^. However, the content of vitamin D in human adipose tissues is generally higher (32–45 µg vitamin D_3_/kg)^(^^25^^–^^27^^)^ than in adipose tissues from pigs found in the present study at 7·6 µg vitamin D_3_/kg. However, content of 200 µg vitamin D_3_/kg in adipose tissue in Göttingen minipigs has been shown following daily exposure to UV light similar to 10–20 min in the midday sun at 55°N during summer^(^^24^^)^.

### Conclusion

This study showed that the concentration and distribution of vitamin D_3_ metabolites (vitamin D_3_ and 25(OH)D_3_) in tissues depend on the ingested form of vitamin D. Furthermore, this study showed that serum 25(OH)D_3_ in pigs is a poor biomarker for the tissue vitamin D_3_ content if the dietary vitamin D source contains both vitamin D_3_ and 25(OH)D_3_.
